# Similar Recovery Rate for Patients Aged between 50 and 89 Years That Go Home on the Surgery Day and Self-Administer Their Rehabilitation after Kinematically Aligned Total Knee Arthroplasty

**DOI:** 10.3390/jpm14050504

**Published:** 2024-05-10

**Authors:** Muzammil Akhtar, Stephen M. Howell, Alexander J. Nedopil, Maury L. Hull

**Affiliations:** 1 College of Medicine, California Northstate University, Elk Grove, CA 95757, USA; 2 Department of Biomedical Engineering, University of California Davis, Davis, CA 95616, USA; sebhowell@mac.com (S.M.H.); mlhull@ucdavis.edu (M.L.H.); 3 Orthopädische Klinik König-Ludwig-Haus, Lehrstuhl für Orthopädie der Universität, 97074 Würzburg, Germany; nedopil@me.com; 4 Department of Orthopedic Surgery, University of California Davis Medical Center, Sacramento, CA 95817, USA; 5 Department of Mechanical Engineering, University of California Davis, Davis, CA 95616, USA

**Keywords:** unrestricted, kinematic alignment, self-administered rehabilitation

## Abstract

Background: for kinematic alignment (KA) total knee arthroplasty (TKA), it was unknown whether ‘the pace of recovery’ at six weeks was different for patients with ages ranging between 50–59, 60–69, 70–79, and 80–89 years who were discharged on the surgery day and self-administered their rehabilitation. Methods: a single surgeon treated 206 consecutive patients with a KA-designed femoral component and an insert with a medial ball-in-socket, lateral flat articulation, and PCL retention. Each filled out preoperative and six-week Oxford Knee Score (OKS), Knee Society Score (KSS), Knee Function Score (KFS), and Knee Injury and Osteoarthritis Outcome Score for Joint Replacement (KOOS, JR) questionnaires. The six-week minus preoperative value indicated improvement. Results: between age cohorts, the improvement was not different (*p* = 0.2319 to 0.9888). The mean improvement/six-week postoperative value was 6°/−2° for knee extension, 0°/119° for knee flexion, 7/31 for the OKS, 39/96 for the KSS, 7/64 for the KFS, and 13/62 for the KOOS. The 30-day hospital readmission rate was 1%. Conclusion: surgeons who perform KA TKA can counsel 50 to 89-year-old patients that they can be safely discharged home on the surgery day with a low risk of readmission and can achieve better function at six weeks than preoperatively when performing exercises without a physical therapist.

## 1. Introduction

Patients of any age who have undergone total knee arthroplasty (TKA) may feel anxious about being discharged home on the same day as their surgery, as well as being unsure about the effectiveness of self-administering their rehabilitation and the risk of readmission. It is commonly believed that younger patients recover more quickly, while older patients, especially those in their eighties, have a slower pace of recovery, a higher risk of readmission, and are more dependent on the care of physical therapists [1,2,3]. However, other studies have shown that older patients have better preoperative overall pain and function scores and better post-TKA outcomes than younger patients [4,5]. Therefore, further research is needed to investigate the impact of age, particularly the differences between patients aged 50–59, 60–69, 70–79, and 80–89 years, on the pace of recovery.

Multiple studies have raised doubts regarding the necessity of formal, supervised, outpatient physical therapy after primary TKA. In some cases, self-administered rehabilitation has been found to be effective, leading to the discontinuation of outpatient physiotherapy in certain preselected patient groups [6,7,8]. However, it remains unclear whether self-administered rehabilitation is effective in patients aged 50–89 years, especially when only brief therapy instructions can be provided because discharge is scheduled on the same day as the surgery.

There is a concern regarding the likelihood of readmission within 30 days after being discharged from the hospital on the day of surgery, particularly in older patients. Studies indicate that individuals over the age of 75, females, those with an ASA score of 4, and those with a body mass index over 35 kg/m^2^ are more likely to experience complications or require an inpatient stay [9]. While there have been reports on the recovery rates and hospital admission rates within 30 days for younger and older patients undergoing mechanical alignment (MA) TKA [10,11], no studies have assessed these issues for kinematically aligned (KA) TKA using a standard protocol of discharge on the day of surgery and self-administering their rehabilitation, which was initiated in July 2020 in response to the COVID-19 epidemic.

This study aimed to examine a group of patients whom a single surgeon treated with a KA TKA and a KA-designed femoral component. The goal was to determine if there were any differences in the patients’ recovery rates, as measured by the improvement in knee extension and flexion, Oxford Knee Score (OKS, with 48 being the best score and 0 being the worst), Knee Society Score (KSS, with 100 being the best score and 0 being the worst), Knee Function Score (KFS, with 100 being the best score and 0 being the worst), and KOOS Jr Score (with 100 being the best score and 0 being the worst), between preoperative and six-week follow-up. Additionally, the study aimed to determine if there was a difference in the incidence of 30-day readmission among patients who were between 50–59, 60–69, 70–79, and 80–89 years of age.

## 2. Materials and Methods

Since 2006, the senior author has performed KA TKA with manual instruments on over 7000 consecutive patients and restored their pre-arthritic alignment regardless of the severity of the preoperative varus, valgus, and flexion deformity. Each patient fulfilled the Centers for Medicare & Medicaid Services’ guidelines for medical necessity for TKA treatment after failing a series of non-operative treatments of nonsteroidal anti-inflammatories (NSAIDs) or analgesics, supervised physical therapy, flexibility and muscle strengthening exercises, assistive device use, weight reduction, and therapeutic injections into knee. Each had radiographic Kellgren–Lawrence Grade III or IV osteoarthritis.

Between November 2021 and May 2023, the author began receiving a limited inventory of a new KA-designed femoral component. The femoral component features a prosthetic trochlear groove with a lateral orientation of 20° relative to a line perpendicular to the distal femoral joint line instead of 6° [12]. After obtaining approval from an institutional review board (Pro00074702), one author (MA) performed a retrospective analysis of a prospective database and identified all patients (N = 206) who received the KA-designed femoral component based on the intraoperative inventory having the patient’s correct size. We excluded those with prior fractures of the knee, inflammatory or septic arthritis, or lower extremity neurologic disorders. Each was treated with cemented components using an insert with a medial ball-in-socket with flat lateral articulation conformity, implanted with posterior cruciate ligament retention (GMK SpheriKA, Medacta International, Castel San Pietro, Switzerland, www.medacta.com, accessed on 5 November 2023) [12,13,14,15].

On the day of the initial consultation but before seeing the surgeon, each patient filled out the Oxford Knee Score (OKS, 48 best and 0 worst), Knee Society Score (KSS, 100 best and 0 worst), Knee Function Score (KFS, 100 best and 0 worst), and KOOS Jr Score (100 best and 0 worst) questionnaires and provided patient demographics on an iPad. In addition, extension, flexion, and alignment deformity of the knee were measured with a long-arm goniometer by the physician’s assistant and added to the iPad. Software uploaded all information to an online HIPAA-compliant database (Caresense, Blue Bell, PA, USA, https://www.caresense.com/ accessed on 1 April 2024). Each patient received a guidebook detailing the perioperative treatment.

Before surgery, each patient was responsible for obtaining medical clearance from their primary care physician and specialists, who assessed their overall medical condition and nutritional status. They also had to enlist a designated postoperative caregiver or ‘coach’, attend a 2 h preoperative class at the hospital with nursing and physical therapy speakers, and make arrangements for their care at home during the first three weeks of recovery.

On the day of surgery, patients received preoperative intravenous medications consisting of Ancef 1–2 gm, Tranzemic Acid 1 gm, Dexamethasone 10 mg, and Ketorolac 30 mg. An anesthesiologist administered general anesthesia to the patient and recorded their ASA physical status. They chose general anesthesia over spinal anesthesia for a few reasons. Firstly, patients could be discharged more quickly from the recovery room because they would not have to wait until they regained control of their legs. Secondly, there was a very low risk of urinary retention in men.

The surgical technique relied upon manual instruments and caliper verification to align the components to resurface the patient’s pre-arthritic knee through a mid-vastus approach with a reported accuracy greater than robotics and a negligible learning curve for the inexperienced surgeon [16,17,18]. A mid-vastus approach was used, which might be a better option than the medial parapatellar approach for primary TKA, as it helps maintain the integrity of the quadriceps muscles and facilitates early recovery [19]. The surgeon injected 50 ccs of 0.2% ropivacaine into the distal femur’s anterior, medial, and lateral periosteum with an 18-gauge spinal needle within 3 min of the incision, which supplemented the general anesthetic and lowered the respiratory rate and heart rate. For the setting of the femoral component, the internal–external (I–E) and varus–valgus (V–V) rotations and the anterior–posterior (A–P) and proximal–distal (P–D) positions were set coincident with the native distal and posterior joint lines by adjusting the calipered thicknesses of the distal and posterior femoral resections to within 0 ± 0.5 mm of those of the femoral component condyles after compensating for cartilage wear and kerf of the saw blade. For the setting of the tibial component, the knee was balanced by adjusting the P–D position, V–V rotation, and medial slope of the tibial resection to match the patient’s pre-arthritic tibial joint line. Best-fitting the anatomic baseplate parallel to the cortical rim of the tibial resection set I–E rotation [20]. The optimum insert thickness satisfied the criteria of maximizing passive external tibial orientation in extension and internal tibial orientation in 90° flexion as measured by an insert goniometer [14]. After making the femoral and tibial resections, the posterior medial and lateral femur were injected with 20 cc of 0.2% ropivacaine using a 20-gauge spinal needle to reduce injury to neurovascular structures. The final injection of 20 cc was administered intra-articularly after closing the extensor mechanism. The use of peripheral nerve blocks was not permitted.

A waterproof and translucent dressing (Opsite Postop Visible, Smith + Nephew, Inc. Memphis, TN, USA) was applied in the operating room. It was left on for seven days, during which the patient was allowed to shower. Following this, the patient applied two 4-inch by 8-inch dressings (Adhesive Island Dressing 7548, Covidien—Medtronic, Inc., Minneapolis, MN, USA) until the staples were removed at 12–14 days.

After leaving the recovery room, patients ate lunch, and a physical therapist provided instructions on self-administering active range of motion (ROM) exercises, walking with a walker, and going up and down stairs. The first two weeks after surgery, instructions were to elevate the knee above the heart on a 10-inch wedge pillow (DMI Ortho Bed Wedge Elevated Leg Pillow, www.amazon.com, accessed on 1 April 2024), and when awake, get up hourly, walk, practice knee range of motion exercises for a few minutes, discard the walker when confident, and strive for 0–90 degrees of motion by two weeks.

Several perioperative conditions did not enable the measurement of blood loss. First, a thigh tourniquet was inflated before the incision and remained elevated until the dressing was applied. Second, the surgeon did not insert an intra-articular drain. Finally, the 6 h time frame between the surgery and the patient’s discharge was too short to detect any significant changes in hemoglobin levels through a blood test.

At six weeks, they filled out the same preop questionnaires on the iPad before seeing the physician’s assistant, who measured the preoperative and postoperative active knee extension and flexion with a long-arm goniometer. One author reviewed the electronic medical records to determine whether and why a patient was readmitted or seen in the emergency room and discharged and whether they had a manipulation under anesthesia (MUA) for stiffness.

A one-way ANOVA approximation calculated the sample size, or number of patients needed to fill each of the 50–59, 60–69, 70–79, and 80–89 age cohorts using a 5° difference in the improvement in knee extension and flexion as the primary dependent variables of the pace of recovery (G*Power, Version 3.1.9.6 access date 21 November 2023) and a 4º standard deviation. Consequently, a group of 22 patients was the smallest sample size for each of the five cohorts (i.e., 110). Because of the possibility of an unequal allocation of patients between cohorts, 206 patients were analyzed. Software determined the mean ± standard deviation (SD) for dependent variables with normal distributions using a goodness-of-fit test for each variable describing the pace of recovery (JMP Pro, 16.0.0, http://www.jmp.com, access date 2 December 2023). A Fisher‘s exact test determined the difference in the proportion of patients with an ASA category, sex, and sidedness between age cohorts. A one-way analysis of variance (ANOVA) determined the significance of the difference in the improvement in BMI, knee flexion and extension, OKS, KSS, KFS, and KOOS Jr, as measured by the six-week value minus the preoperative value, between the four age cohorts. A significant difference was *p* < 0.05.

## 3. Results

The proportion of the 206 patients in each age cohort was 15% (31) in 50–59, 38% (79) in 60–69, 35% (71) in 70–79, and 12% (25) in 80–89. The preoperative sex distribution, knee sidedness, BMI, ASA score, knee flexion, extension, OKS score, KSS score, KFS score, and KOOS Jr. score were not significantly different between age cohorts (*p* = 0.1165 to 0.9806) (Table 1).

The pace of recovery, as measured by the six-week minus the preoperative value, for knee flexion, extension, OKS, KSS, KFS, and KOOS Jr, was not significantly different between age cohorts, which allowed pooling of the data (*p* = 0.2319 to 0.9888) (Table 2). The pooled mean improvement/six-week postop values for all patients were 6°/−2° for knee extension, 0°/119° for knee flexion, 7/31 for the OKS, 39/96 for the KSS, 7/64 for the KFS, and 13/62 for the KOOS Jr. The physician’s assistant encouraged patients to schedule a second visit when they were dissatisfied with the speed of their recovery and their level of improvement. However, 90% of patients declined to schedule a second visit.

Two patients required readmission, and four presented to the ER within 30 days of the index KA TKA (Table 3). One 57-year-old female patient underwent an MUA for lack of flexion past 105°.

## 4. Discussion

The most important findings were that patients who undergo KA TKA and follow a standard protocol of being discharged to home on the surgery day and performing self-administered rehabilitation can expect a similar pace of recovery regardless of their age (50 to 89 years). Surgeons can counsel their patients regarding this. Furthermore, patients can expect better motion and PROMs at six weeks post-surgery than in their preoperative state. The rate of hospital readmission and emergency room visits in the first 30 days post-surgery is low, at 1% and 2%, respectively.

The present study’s result, which indicates that patients aged 50 to 89 have a similar pace of recovery after KA TKA, agrees with and contradicts findings from other studies. For example, in a multicenter retrospective study of MA TKA, the authors found that at six weeks the KSS pain and motion scores were significantly higher in the >75-year-old group relative to the 55 to 74 and the <55 groups. The authors attributed the variability in postoperative PROMs to higher preoperative expectations in younger patients and a higher number of comorbidities in older patients [11]. In contrast, another retrospective study of MA TKA showed that the <80-year-old group had significantly higher KSS function scores at six weeks and comparable overall KSS scores when compared with the ≥80-year-old group [2]. An explanation for the comparable pace of recovery after KA between ages 50 and 89 years in the present study is KA’s reported low morbidity from not releasing ligaments, which might have provided better pain relief and early mobility, decreased length of stay, and resulted in a more normal feeling knee relative to MA [2,10,21,22].

In July 2020, the hospital’s response to the COVID-19 pandemic was to discharge all patients treated with primary TKA on the surgery day regardless of age, ASA physical status, and pre-morbidities, which, after 32 months, is a process that remains unchanged and functions well. The success across all age groups in the present study, in which 41% were ASA 3 and 53% ASA 2, contrasts with those studies showing that the optimal patients for discharge on the surgery day are generally younger ones with a low ASA classification [9,23]. Some explanations for the program’s success include a comprehensive 2 h preoperative class offered by the hospital with the education provided by nursing and physical therapy, clearance from primary care and specialty physicians, perioperative pain management consisting of a 90 cc local analgesic infiltration of the femoral periosteum and intra-articular space that lasts 30 h, and the insistence that the patient arranges for a ‘coach’ to assist them during the first two weeks.

Many studies have shown that a home program of self-administered rehabilitation is non-inferior to outpatient physical therapy, and the present study supports this conclusion [6,7,8]. In the present study, the 0.5% incidence of MUA after performing self-administered rehabilitation in a 57-year-old female patient was low compared to the 3% incidence reported for a home exercise program and outpatient physical therapy in a study of MA TKA. The cost savings are substantial, as eliminating outpatient physical therapy is reported to save $1341 for Medicare patients and $1893 for those with private insurance [6]. A final benefit of self-administered rehabilitation is that it eliminates the disadvantage of the patient finding someone to drive them to the treatment. However, surgeons should consider that some select patients may still require formal physical therapy, but most do not.

The low risk of hospital readmission (1%) and emergency room visits (2%) in the first 30 days in the present study suggests that discharge to home on the surgery day did not result in a high incidence of unexpected costs, regardless of patient age and ASA score. The present study of the KA TKA 30-day readmission rate was low when compared to a retrospective, propensity-matched cohort study of patients ≥ 80 years of age treated with MA TKAs that had a 3.5% readmission rate and complication rates that were similar for the 709 patients discharged on the surgery day and the 709 inpatients [1]. However, a national database study of MA TKA found that an ASA score ≥3 was a more prominent risk factor for adverse postoperative events [3]. Therefore, healthcare providers should consider this factor when advising patients to consider being discharged home.

The present study has certain limitations that need to be considered. Firstly, the patients who participated in the study might not represent the motivation and determination of all patients who undergo TKA. This is because some patients who are worried about being discharged on the day of surgery or concerned about the effectiveness of self-administered rehabilitation exercises may opt for care elsewhere. Secondly, patients who underwent KA TKA below the age of 50 were not included in the study due to the small number of patients in that age group. The calculated smallest cohort sample size of 22 was not met. Thirdly, postoperative motion and PROMs were only obtained six weeks post-surgery, representing a short follow-up period. However, it is important to note that the aim of the present study was not to compare patients’ recovery after extended periods. Fourthly, a Type II error may be caused by the sample size in each age group. However, it is unlikely that a larger sample size would detect differences in the mean OKS, KSS, KFS, and KOOS JR between age cohorts (Table 2) since the mean differences were smaller than the reported minimal clinically important difference of six for the OKS, seven for KSS, six for KFS, and nine for the KOOS JR.

## 5. Conclusions

The results of this study suggest that surgeons who perform caliper-verified KA TKA using manual instruments and a KA-designed femoral component and insert with a medial ball-in-socket and lateral flat articulation, PCL retention, and use an insert goniometer to select the optimal thickness can counsel 50 to 89-year-old patients that regardless of age, they can be safely discharged home on the surgery day with a low risk of readmission, can administer their rehabilitation at home without the supervision of a physical therapist, and can expect a rapid pace of recovery with a level of function at six weeks postoperatively that is better, on average, than preoperatively. Further study is required to determine whether other alignment strategies and implant designs can match these results.

## Figures and Tables

**Table 1:** Patient Demographics and Preoperative Characteristics for Each Age Cohort.

	50–59 (n = 31)	60–69 (n = 79)	70–79 (n = 71)	80–89 (n = 25)	*p*-Value
Demographics and Operative Characteristics					
Sex (Male/Female)	12/19	39/40	31/40	11/14	0.7654
Side of KA TKA (Right/Left)	16/15	42/37	37/34	12/13	0.9806
Age (Years)	56 ± 3.1	65 ± 2.9	74 ± 2.8	83 ± 2.2	N/A
Body Mass Index (kg/m^2^)	31 ± 5.5	29 ± 5.3	29 ± 5.3	28 ± 4.8	0.2370
ASA Score (1/2/3/4/5)	2/21/5/0/0	3/40/31/0/0	4/32/29/1/0	1/8/12/0/0	0.1240
Operative Time (minutes)	56 ± 10	56 ± 11	54 ± 9	53 ± 8	0.1244
Preoperative Range of Motion, Function, and Kellgren–Lawrence Classification of Osteoarthritiis					
Flexion	120 ± 9.5	119 ± 9.4	119 ± 8.3	120 ± 7.1	0.8659
Extension	11 ± 7.5	9 ± 7.1	8 ± 6.5	8 ± 6.8	0.2654
OKS (48 best, 0 worst)	22 ± 10	24 ± 9.1	25 ± 8.7	22 ± 7.8	0.2895
KSS (100 best, 0 worst)	56 ± 28.3	57 ± 26.2	59 ± 28.5	58 ± 33.5	0.9609
KFS (100 best, 0 worst)	57 ± 30.4	59 ± 21.8	59 ± 22.3	47 ± 19.5	0.1165
KOOS JR (100 best, 0 worst)	45 ± 15	50 ± 16.1	51 ± 13.4	47 ± 10.5	0.2820
Kellgren–Lawrence Classification	III, n = 10 IV, n = 21	III, n = 33 IV, n = 46	III, n = 30 IV, n = 41	III, n = 8 IV, n = 19	0.3562

Reported as mean ± standard deviation. N/A, not applicable.

**Table 2:** Pace of Recovery at Six Weeks for Each Characteristic for Age Cohorts.

	50–59 (N = 31)	60–69 (N = 79)	70–79 (N = 71)	80–89 (N = 25)	*p*-Value
Pace of Recovery Characteristic					
Flexion	−4 ± 16	1 ± 13	1 ± 14	0 ± 10	0.2319
Extension	7 ± 10	7 ± 7	6 ± 6	4 ± 6	0.3420
OKS (48 best, 0 worst)	9 ± 12	6 ± 10	7 ± 11	8 ± 9	0.7287
KSS (100 best, 0 worst)	38 ± 29	40 ± 27	39 ± 28	40 ± 32	0.9888
KFS (100 best, 0 worst)	12 ± 22	7 ± 23	5 ± 19	10 ± 17	0.3452
KOOS JR (100 best, 0 worst)	17 ± 18	12 ± 8	13 ± 15	14 ± 11	0.5623

Reported as mean ± standard deviation.

**Table 3:** Demographics and Reasons for Readmission or Emergency Room Visit.

30-Day Readmission or Emergency Room Visit	Age	Sex	ASA	Reason
Readmission	74	M	3	Superficial femoral vein thrombosis
Readmission	61	M	3	Acute delirium, acute hypoxic respiratory failure, hyponatremia, essential tremor, paroxysmal atrial fibrillation
Emergency Room Vist	85	M	3	TKA and lower extremity pain
Emergency Room Vist	75	F	4	TKA and lower extremity pain
Emergency Room Vist	78	M	3	TKA and lower extremity pain
Emergency Room Vist	50	F	2	High blood pressure, lower extremity spasms, migraines

## Data Availability

The raw data supporting the conclusions of this article will be made available by the authors (sebhowell@mac.com) on request.
